# Travel burden and carbon dioxide emission reductions through a model of cancer care closer to the patient

**DOI:** 10.1093/oncolo/oyaf021

**Published:** 2025-02-18

**Authors:** Luigi Cavanna, Chiara Citterio, Costanza Bosi, Stefano Vecchia, Manuela Proietto, Patrizia Mordenti

**Affiliations:** Casa di Cura Piacenza, 29121 Piacenza, Italy; Oncology-Haematology Department, AUSL Piacenza, 29121 Piacenza, Italy; Oncology-Haematology Department, AUSL Piacenza, 29121 Piacenza, Italy; Pharmacology Department, AUSL Piacenza, 29121 Piacenza, Italy; Oncology-Haematology Department, AUSL Piacenza, 29121 Piacenza, Italy; Oncology-Haematology Department, AUSL Piacenza, 29121 Piacenza, Italy

**Keywords:** cancer patients, travel burden, carbon dioxide emission, territorial oncology

## Abstract

**Background:**

Traveling to healthcare facilities, particularly in the case of patients requiring frequent visits and repeated treatments, such as cancer patients, is associated with substantial carbon dioxide (CO_2_) emissions. Moreover, travel burdens can delay diagnosis and negatively influence prognosis.

**Materials and Methods:**

In 2004, a programme called territorial oncology care (TOC) was initiated in the province of Piacenza (northern Italy) to relieve travel burdens by providing treatment closer to patients’ residences. Patient management is performed by oncologists traveling from the city hospital’s oncologic ward to rural centers. Patients are managed at 3 rural hospitals and a health center called Casa della Salute. In this study, we retrospectively analyzed all files containing the schedules of patients enrolled in the TOC programme from 2 January 2017 to 31 December 2022. We calculated the driving distance (in kilometers) to the outpatient facility closest to each patient’s residence and the CO_2_ emissions saved compared with the distance to the city hospital.

**Results:**

A total of 2132 cancer patients on active treatment were enrolled in the TOC programme during the study period. The total travel saved by treating patients closer to their residences over this 6-year period amounted to 1 975 105 km, representing carbon emission savings of 241.56 tonnes.

**Conclusion:**

Our findings show a significant reduction (241.56 tonnes) of CO_2_ emissions for the entire cohort of patients over a period of 6 years.

Cancer patients’ travel burdens and associated carbon emissions can be substantially reduced by programmes such as TOC.

## Introduction

Patients with cancer must overcome many barriers of economic, psychological, social, and even of familial nature.^1^ The distance from a patient’s residence to a treatment center may represent an additional obstacle that can delay diagnosis and influence management, interfering with evidence-based medicine.^1-3^ Several studies have shown that cancer patients who travel long distances for treatment may experience worse prognosis and greater impairment in quality of life, with many of these studies focusing on travel distances, time toxicity, and the impact of travel burdens on diagnosis delays and outcomes. However, traveling to healthcare facilities, above all in the case of patients requiring frequent visits and repeated treatments as cancer patients, is associated with carbon dioxide (CO_2_) emission.^4^

To reduce travel burdens and their consequences for patients with cancer, in 2004, the Oncology and Haematology Department of Piacenza’s hospital (northern Italy) initiated a programme called territorial oncology care (TOC) aimed at delivering oncologic management closer to patients’ residences.^3^

In this retrospective study of 2132 patients, we aimed to evaluate the reduction in patients’ travel burdens (in terms of distances traveled) and the consequent reduction in CO_2_ emissions achieved over a 6-year period through an innovative patient-centered programme based on decentralized healthcare and anticancer infusion treatment provided closer to patients’ residences.

## Materials and methods

To promote patient-centered care, to reduce travel times and the associated burdens for cancer patients in the province of Piacenza, that comprises rural and mountaineers area and has a population of ~286 000 individuals, the Oncology and Haematolgy Department, hospital of Piacenza, established the TOC programme in 2004. This model of care for oncologic patients includes outpatient clinics in 3 rural community hospitals, and as of July 2016 in a health center called Casa della Salute (CdS) (Supplementary Figure S1). Outpatient management is provided in dedicated areas in the 3 rural hospitals and CdS by oncologists who travel there from the City Hospital of Piacenza by public transit. This model provides the same modality of care as that provided by the referral hospital since cancer patients are managed by the same specialists (oncologists and specialized nurses). All cases treated through the TOC programme are discussed in a multidisciplinary fashion at the oncology department of the referral hospital to share therapeutic choices and to increase the possibility of experimental clinical trials involving these patients. After the multidisciplinary evaluation, the patient is informed about the selected treatment for her/him, and she/he can chose the site of medical treatment: near the residence or to the city referral hospital. All systemic treatment given at the city referral oncology outpatient clinic were done at the community centers.

In this study, we retrospectively analyzed all the electronic files containing the schedules of patients managed in the TOC program from January 2, 2017 to December 31, 2022. These files contain demographic and clinical data, such as sex, age, type and stage of cancer, type and line of treatment, number of visits, and number of infusion therapy administrations. Using Google Maps, we calculated the driving distance (in kilometers) to the outpatient service closer to each patient’s residence and the kilometers and time saved compared with the distance to reach the referral city hospital. Furthermore, we evaluated the CO_2_ emissions per kilometer traveled by private transport according to the values provided by the European Environment Agency for the period 2017-2022 (122.3 g of CO_2_ per kilometers).^5^ The calculation was based on travel by personal car, as it is by far the most common mode of transportation for patients in our district. This study was approved by the Institutional Review Board (or Ethics Committee) of AVEN (Area Vasta Emilia Nord) (Protocol n. 2023/0040653 12/04/2023).

### Statistical analysis

Quantitative variables were expressed as medians and interquartile ranges (IQRs), while qualitative variables were expressed as absolute and relative frequencies. All analyses were performed using RStudio statistical software (version 3.6.0).

### End points

The primary end point of interest was the carbon emission savings associated with the TOC programme each year over the 6-year study period. The secondary end point was the reduction in travel burdens (in terms of kilometers traveled) for the patients.

## Results

The clinical and demographic characteristics of the 2132 cancer patients enrolled in the TOC programme from January 2, 2017 to December 31, 2022 and undergoing anticancer therapy are reported in Table 1. Among these patients, 1109 (52.02%) were women. The patients’ median (IQR) age was 71 (63-73) years (range 28-92 years). The types of cancer were gastrointestinal (40.10%), lung (19.51%), breast (18.86%), genitourinary (14.49%), head and neck (2.53%), and other (4.40%). Most patients (78.19%) had metastatic disease, while 465 (21.81%) had localized early-stage cancer.

**Table 1.** Demographic and clinical characteristics of the 2132 cancer patients enrolled in the TOC programme from 2017 to 2022.

Variable	Value	Year					
		2017 (*n* = 278)	2018 (*n* = 347)	2019 (*n* = 354)	2020 (*n* = 360)	2021 (*n* = 379)	2022 (*n* = 414)
Sex, *n* (%)							
Female	1109 (52.02)	144 (51.80)	173 (49.86)	190 (53.67)	190 (52.78)	193 (50.92)	219 (52.90)
Male	1023 (47.98)	134 (48.20)	174 (50.14)	164 (46.33)	170 (47.22)	186 (49.08)	195 (47.10)
Age, median [IQR] (range)	71 [63-73] (28-92)	66 [59-71] (30-86)	67 [60-72] (28-87)	67 [61-73] (34-91)	62 [72-75] (35-92)	70 [62-76] (30-88)	71 [63-77] (31-90)
Anatomical location, *n* (%)							
Gastrointestinal	855 (40.10)	124 (44.55)	160 (46.09)	144 (40.74)	134 (37.33)	139 (36.68)	154 (37.20)
Genitourinary	309 (14.49)	27 (9.57)	41 (11.73)	51 (14.29)	64 (17.87)	59 (15.57)	68 (16.43)
Breast	402 (18.86)	45 (16.17)	64 (18.44)	67 (19.05)	65 (18.13)	76 (20.05)	85 (20.53)
Lung	416 (19.51)	66 (23.76)	68 (19.55)	74 (20.90)	68 (18.93)	70 (18.47)	70 (16.91)
Head and neck	54 (2.53)	9 (3.30)	8 (2.23)	5 (1.32)	5 (1.33)	15 (3.96)	13 (3.14)
Other	94 (4.40)	7 (2.64)	7 (1.96)	13 (3.70)	23 (6.40)	20 (5.28)	24 (5.80)
Stage, *n* (%)							
Localized	465 (21.81)	62 (22.30)	87 (25.07)	78 (22.03)	73 (20.28)	88 (23.22)	77 (18.60)
Metastatic	1667 (78.19)	216 (77.70)	260 (74.93)	276 (77.97)	287 (79.72)	291 (76.78)	337 (81.40)
Type/line of therapy, *n* (%)							
Neoadjuvant/adjuvant	465 (21.81)	62 (22.30)	87 (25.07)	78 (22.03)	73 (20.28)	88 (23.22)	77 (18.60)
First-line	980 (45.97)	119 (42.82)	150 (43.23)	156 (44.07)	170 (47.22)	171 (45.12)	214 (51.69)
Second-line or subsequent	686 (32.17)	97 (34.89)	110 (31.70)	120 (33.90)	117 (32.50)	120 (31.66)	122 (29.47)

Abbreviation: IQR, interquartile range.

### Primary end point

The treatment of cancer patients closer to their residences resulted in carbon emission savings of 31.86 tonnes in 2017, 37.81 tonnes in 2018, 38.06 tonnes in 2019, 40.51 tonnes in 2020, 44.31 tonnes in 2021, and 49.01 tonnes in 2022. The total emissions saved over the 6-year period amounted to 241.56 tonnes (Table 2).

**Table 2.** Number of visits, infusion therapy, ktabilometres, and tonnes of CO_2_ saved by treating cancer patients near their residences each year and over the entire 6-year study period.

Year	Number of patients	Number of visits	Number of infusion therapy administrations	Km saved per patient	Total km saved	CO_2_ emissions avoided (tonnes)
2017	278	2214	3897	937	260 486	31.86
2018	347	2652	4323	891	309 177	37.81
2019	354	2524	4152	879	311 166	38.06
2020	360	2613	4290	920	331 200	40.51
2021	379	2254	3884	956	362 324	44.31
2022	414	2541	4088	968	400 752	49.01
Total	2132	14 798	24 634	5551	1 975 105	241.56

### Secondary end point

The total kilometers saved were 260 486 in 2017 (278 patients), 309 177 in 2018 (347 patients), 311 166 in 2019 (354 patients), 331 200 in 2020 (360 patients), 362 324 in 2021 (379 patients), and 400 752 in 2022 (414 patients). A total of 1 975 105 km were saved over the 6-year period (Table 2). The median distance from the patient’s residence to the city hospital was 26 kms (range 11-79 kms), while it was 7 kms (range 1-35 kms) to the community hospital and CdS; the median time to reach the city hospital was 44 minutes (range 32-116) versus 16 minutes (range 16-23) to the community hospital and CdS.

## Discussion

Healthcare services significantly contribute to CO_2_ emissions, accounting for 4%-5% of total global emissions.^4^ Transportation to healthcare facilities, especially for patients with diseases requiring repeated visits and infusion therapies, such as cancer patients, is associated with substantial carbon emissions.^4^ Many patients with metastatic cancer live considerably longer than in the past, thanks to new drugs. However, they require prolonged treatment with anticancer and supportive drugs. Routine clinic appointments represent substantial burdens and times not only for visits and treatments but also for travel, parking, and other transit needs. Disparities in resource allocation can affect access to care, as patients may need to travel long distances to receive treatment.^6,7^

Treating cancer patients closer to their homes can also significantly reduce carbon emissions. In our district 98.90% of cancer patients chose to be treated closer to their residence as previously reported.^3^ In our district, the vast majority of patients with cancer living in the city of Piacenza are treated in the city hospital and the vast majority of patients living in the rural area are treated in the TOC program. In 2020, the UK National Health Service adopted formal plants to transition from being responsible for 4% of the UK’s carbon emissions to achieving net zero emissions by 2040.^8^ To reach this goal, changes to healthcare practices are needed at many levels, including patient travel.^9^ We believe 240 tons of CO₂ is a significant value, approximately equivalent to 2 full long-haul flights or one and a half flights of a very large aircraft, such as a Boeing 777 or an Airbus A380. The Global Burden of Diseases, Injuries, and Risk Factors study found that among the risk factors analyzed, atmospheric particulate matter was the leading contributor to the global disease burden in 2021.^10^

Nevertheless, in rural areas, anticancer therapy can be provided closer to patients’ residences, as has been done in our province since 2004.^3,9^ Physicians and healthcare decision-makers should bear in mind that the travel burdens experienced by patients and their relatives often affect their quality of life, reduce the time spent at home, increase costs, and have an environmental impact, which has been hitherto overlooked.^1-3,9^ However, our study has some limitations: it is retrospective and monocentric. This territorial model could count on pre-existing peripheral hospitals that allowed the realization of the TOC program.

## Conclusion

To our knowledge, this is the first study to quantify the environmental benefit of providing cancer treatment closer to patients, based on a large series of 2132 patients, which is representative of a real-world population treated in an oncologic department. The study focuses solely on the savings related to patient travel, as the project was initially designed with the goal of reducing the travel burden. Our findings show a significant reduction (241.56 tonnes) of CO_2_ emissions for the entire cohort over a period of 6 years. We believe that cancer treatment closer to patients is feasible and can reduce CO_2_ emissions, travel burden, and time toxicity.

## Supplementary Material

oyaf021_suppl_Supplementary_Figures_1

## Data Availability

Data available on request from the authors.
