# Supplementary material for: Travel burden and carbon dioxide emission reductions through a model of cancer care closer to the patient
Source: Oncologist. 2025 Feb 18;30(2):oyaf021. doi: 10.1093/oncolo/oyaf021 (PMC11833240; doi:10.1093/oncolo/oyaf021)

Supplemental Figure 1: TOC program in the province of Piacenza: oncology care sites after the addition of the Val Nure site in Bettola in July 2016. (TOC territorial oncology care)


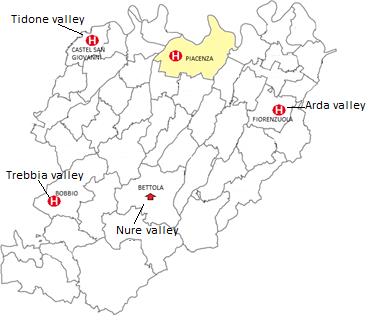

Supplement: oyaf021_suppl_Supplementary_Figures_1 [file oyaf021_suppl_supplementary_figures_1.docx]
